# The prevalence and mental health correlates of exposure to offensive behaviours at work in Hungary: results of a national representative survey

**DOI:** 10.1186/s12889-022-14920-0

**Published:** 2023-01-11

**Authors:** Tyler Szusecki, Barna Konkolÿ Thege, Adrienne Stauder

**Affiliations:** 1 Waypoint Research Institute, Waypoint Centre for Mental Health Care, Penetanguishene, ON Canada; 2 Department of Psychiatry, University of Toronto, Toronto, ON Canada; 3 Semmelweis University, Institute of Behavioral Sciences, Budapest, Hungary

**Keywords:** Workplace bullying, Offensive behaviours, Prevalence, Mental health, Representative sampling, Hungary

## Abstract

**Background:**

Within the last decades, a substantial number of reports have established bullying behaviours as a severe risk to the health and safety of workers. However, in Hungary, the severity of this issue remains largely unknown. Therefore, the current study aimed to 1) determine the prevalence of offensive workplace behaviours in the Hungarian working population and 2) examine the relationship between exposure to these offensive behaviours and certain mental health indicators.

**Methods:**

The cross-sectional analyses of the present study are based on a sample of 13,104 active workers being representative of the Hungarian working population according to gender, age, educational level, and 18 occupational sectors. The mid-length version of the Copenhagen Psychosocial Questionnaire II (COPSOQ II) was used to measure workplace offensive behaviours (bullying, sexual harassment, threats of violence, and physical violence) in the 12 months preceding the survey. Examined mental health correlates included depressive symptomatology (Beck Depression Inventory), functional somatic symptoms (PHQ-15), perceived stress (Perceived Stress Scale), and general well-being (WHO Well-being Index).

**Results:**

Almost half (48.7%) of the sample reported exposure to some form of offensive behaviour; 37.6% of participants reported occasional-, while 11.1% reported weekly or daily exposure. More women than men were exposed to offensive workplace behaviours, and those targeted the most were individuals aged 18–29 and in companies employing 20–49 employees. Top managers reported the lowest amount of bullying, while unskilled labourers reported the most frequent exposure. A moderately strong relationship was discovered between exposure to workplace offensive behaviours and all indicators of mental health.

**Conclusion:**

Workplace bullying was revealed to be a significant public health concern according to this large, representative data set from Hungary. Strategies to reduce the occurrence and impact of these behaviours on employee health should be a priority for occupational health and safety interventions.

**Supplementary Information:**

The online version contains supplementary material available at 10.1186/s12889-022-14920-0.

## Introduction

Within the last three decades, researchers have amassed a body of evidence on the presence of bullying and other harmful behaviours in the workplace that posit the issue as a serious risk to the health and safety of workers [1, 2]. The current literature suggests that exposure to these behaviours may result in serious physical and psychological complications, including symptoms of post-traumatic stress, burnout, reduced job satisfaction, low organizational commitment [3], anxiety, irritability, sleeping problems [4], poor workplace performance [5], depression [6], increased absenteeism [7], and higher rates of cardiovascular disease [8]. However, despite the outstanding evidence, the prevalence of these behaviours has been shown to vary considerably across studies, at least partially as a result of variations in sampling strategies and choice of measurement [9, 10].

Typically, researchers will employ one of two primary methods to estimate the prevalence of workplace bullying; although, in some cases, more than one method is employed [9, 11]. The first method, referred to as the behavioural experience approach, estimates the prevalence of bullying by inviting participants to report how often they have been exposed to various negative social acts in the workplace [11, 12]. An inventory that exemplifies this approach is the Negative Acts Questionnaire, developed by Einarsen and colleagues in 1994 and revised in 2009 as the NAQ-R [13]. The second method, a subjective assessment referred to as the self-labelling approach, invites participants to identify whether or not they have been subjected to bullying in the workplace, where in most cases, a brief definition of the term is provided to the participant prior to this inquiry [12].

When reviewing scientific examinations of workplace bullying, it is imperative to first identify the choice of measurement and sampling strategies employed by researchers and clinicians. Evidence has shown that valid comparisons cannot be made without taking these moderators into account [10], as prevalence rates reported through participant self-labelling methods tend to result in lower values on average, while behavioural experience methods tend to result in higher values on average [14]. It is therefore important to be cautious for the potential of inter- and intra-method variance when making comparisons between studies.

The fifth European Working Conditions Survey serves as a reliable reference for self-labelling methodologies and clearly illustrates an instance of inter-method variance, defined by the potential variance that exists exclusively within each form of measurement. In this survey, it was discovered that exposure to workplace bullying in the European context was most common in France (9.5%), Netherlands (7.7%), Luxembourg (7.2%), and Austria (7.2%), and least common in Bulgaria (0.6%), Poland (0.7%), and Italy (0.9%) [15]. These results were supported by similar findings in the years prior in studies among the likes of Niedhammer and colleagues (2006), who reported a prevalence rate of 8.78% of men and 10.7% of women experiencing workplace bullying in the French working population using similar methods [16].

In recent years, studies have reported prevalence rates as high as 15% in New Zealand [17], 31% in the United States [18], and 11.2% in Malaysia [19], following the behavioural experience method, and demonstrating, by comparison of these aforementioned studies, an example of intra-method variance, defined as the inherent variance that exists between these two forms of measurement. That being said, several studies have reported estimates with industry as a variable of interest, and those observed to be at the highest risk for exposure have largely remained the same when controlled for choice of measurement and sampling demographics. In the literature, they have been defined as educational professionals (14.9% within six months) [20], health care workers (14.3% within the last six months) [21], public administration and defence workers (10.8% within the last six months) [22], accommodation and food service associates (8.2% within the last six months), manufacturing employees (7.8% within the last six months), and construction workers (7.4% within the last six months). As such, studies that concentrate on these sectors will often produce a higher frequency value, on average, and may not be indicative of the problem on a national level.

Without a valid comparison, it may be difficult for researchers to generalize their findings and identify the severity of workplace bullying from low to high, especially when the focus of the study is limited to one particular variable, such as industry, gender, or occupational status [10]. Therefore, in order to obtain an accurate assessment of the severity of workplace bullying, ideally, samples should be representative of the working population, and comparisons should be sensitive to the methodological moderators that have the potential to affect the outcome of such data.

In Hungary, comprehensive evidence on workplace bullying is scarce. The current study is the first of its kind in Hungary, and one of very few in Central and Eastern Europe, to identify vulnerable populations by measuring the prevalence of offensive workplace behaviours in a large, representative sample. Moreover, due to the suboptimal consistency in researchers’ choice of measurement, sampling strategies, and operative criteria surrounding workplace bullying in the literature, valid comparisons between studies have become more challenging to make and, consequently, vulnerable populations more difficult to define. Thus, the primary aim of the current study was to contribute to the scarce body of empirical evidence surrounding the prevalence of workplace bullying in Hungary by reporting the results of a representative study from a sample of eighteen occupational sectors. As a secondary aim, we also intended to examine the relationship between exposure to workplace offensive behaviours and a few common mental health indicators (i.e., depressive symptomatology, functional somatic symptoms, perceived stress, and general wellbeing) to better understand the interrelationship between health and negative workplace experiences.

## Methods

### Subjects and data collection

All procedures performed in this study were in accordance with institutional guidelines and the 1964 Helsinki declaration and its later amendments. The study protocol was approved by the Regional and Institutional Committee of Science and Research Ethics at Semmelweis University (TUKEB No 195/2012). All participants gave written, informed consent prior participation in the study.

Data were collected in Hungary using an online questionnaire accessed via a secure website (www.munkahelyistreszkerdoiv.hu). Several sampling strategies were used simultaneously to increase sample size [23]. Information about the study and access to the online questionnaire were distributed via email lists, social media sites, as well as websites of universities and non-profit organizations. National electronic media also publicized the study through online newspapers, public and commercial television, and radio broadcasts. As an incentive to complete the questionnaire, respondents received immediate automatized feedback comparing their own results with the current national and sector-specific mean scores.

Over the entire data collection period (May 2013 to March 2014), 19,280 individuals gave their consent and started filling in the questionnaire and 13,932 (72.2%) respondents reached the end of the test battery. Eight hundred and twenty-eight questionnaires (6%) were excluded from the data set due to missing data (i.e., more than 20% of items left unanswered on the COPSOQ II, the main questionnaire of the test battery; missing answers regarding demographic variables the weighting was based on) or invalid answers (e.g., males reporting on menstruation-related concerns; unrealistic answers to open ended questions such as more than 90 years worked, more than 16 h of work per day etc.). The data cleaning resulted in a final data set of 13,104 respondents who had paid work for at least 3 months preceding the survey. Demographic characteristics of the sample are summarized in Table 1. Representativity in terms of gender, age, education, and the 18 occupational sectors – based on data from the Hungarian Central Statistical Office [24] – was achieved through a weighting process. Characteristics of the weighted sample are also presented in Table 1. Sample description in terms of occupational sectors can be found in Supplementary Table 1.

**Table 1:** Sociodemographic characteristics of the sample

	Started *N* = 19,280		Completed *N* = 13,932		Included *N* = 13,104		Weighted sample
	*N*	%	*N*	%	*N*	%	%
Gender							
Women	11,637	60.4	8,715	62.6	8,323	63.5	46.3
Men	7,642	39.6	5,216	37.4	4,781	36.5	53.7
Missing	1	0.0	1	0.0	0	0.0	0.0
Age group							
18–29	6,036	31.3	3,967	28.5	3,675	28.0	17.0
30–39	6,346	32.9	4,615	33.1	4,359	33.3	30.6
40–49	3,990	20.7	3,020	21.7	2,857	21.8	26.0
50–59	2,447	12.7	1,976	14.2	1,890	14.4	23.3
≥ 60	460	2.4	353	2.5	323	2.5	3.1
Missing	1	0.0	1	0.0	0	0.0	0.0
Level of education							
Primary (8 years)	242	1.3	139	1.0	105	0.8	10.8
Vocational or technical school (10–12 years)	4,896	25.4	3,445	24.7	3,224	24.6	28.8
High school (12–13 years)	2,852	14.8	1,965	14.1	1,819	13.9	34.0
University or college diploma	11,260	58.4	8,360	60.0	7,956	60.7	26.3
Missing	30	0.2	23	0.2	20	0.2	0.1
Current position							
Unskilled worker	1,330	6.9	870	6.2	780	6.0	15.4
Skilled worker	3,427	17.8	2439	17.5	2,243	17.1	25.6
Leader without diploma	1,043	5.4	757	5.4	719	5.5	10.7
Professional	6,308	32.7	4,584	32.9	4,351	33.2	20.3
Administrative	4,178	21.7	3,058	21.9	2,892	22.1	18.4
Middle manager	2,023	10.5	1,542	11.1	1,483	11.3	6.3
Upper manager	969	5.0	681	4.9	636	4.9	3.3
Missing	2	0.0	1	0.0	0	0.0	0.0
Residence							
Capital	7,943	41.2	5709	41.0	5,374	41.0	31.3
Chief town of a county	3,693	19.2	2725	19.6	2,579	19.7	20.2
Town	5,220	27.1	3,730	26.8	3,509	26.8	32.9
Village	2,423	12.6	1,767	12.7	1,642	12.5	15.6
Missing	1	0.0	1	0.0	0	0.0	0.0
Marital status							
Single	5,796	30.1	3,963	28.4	3,702	28.3	22.2
Common-law partner	4,690	24.3	3,347	24.0	3,132	23.9	22.7
Married	7,009	36.4	5,246	37.7	4,964	37.9	42.7
Divorced	1,552	8.0	1,201	8.6	1,147	8.8	10.8
Widow	232	1.2	174	1.2	159	1.2	1.6
Missing	1	0.0	1	0.0	0	0.0	0.0

### Measures

The sociodemographic section of the test battery included questions regarding the respondents’ gender, age group, educational attainment, residence, marital status, job type (for response options, see Table 1), occupational sector (using the 18 occupational sectors defined by the Hungarian Statistical Office, see Fig. 5), and company size.

To assess the occurrence of offensive behaviours, the middle-length, Hungarian version [25] of the COPSOQ II [26] was used.^1^ This comprehensive assessment tool, using the self-labelling method, covers exposure to four types of workplace-specific offensive behaviours (bullying, sexual harassment, threats of violence, and physical violence) and uses a single item for each of the four domains and a 12-month time frame when inquiring about the occurrence of such experiences. Response options for each item are as follows: No (0), Yes, a few times (1), Yes, monthly (2), Yes, weekly (3), Yes, daily (4). In accordance with the general consensus regarding the assessment of bullying, we formed three frequency categories for overall offensive behaviour exposure: no exposure, occasional exposure (if the frequency of any of the four offensive behaviours was monthly or less frequent) and frequent exposure (if the frequency of any of the four offensive behaviours was weekly or daily). If the respondent reported their exposure to a given offensive behaviour, a follow-up question was asked about the identity of the perpetrator with the following response options: colleague, manager / superior, subordinate, client / customer / patient.

Depressive symptoms were measured with the shortened Hungarian version [27] of the Beck Depression Inventory (BDI) [28], a 9-item questionnaire to assess depression symptom severity. Each item is scored on a 4-point scale ranging from 0 (not at all characteristics of me) to 3 (very characteristic of me). Internal consistency of the scale proved to be very high in the current sample (Cronbach’s alpha = 0.87) (Cronbach’s alpha values across the manuscript have been interpreted based on [29]). To allow for international comparisons, the total score of the 9-item version was transformed to align with the 21-item original version by multiplying the total scores by 2.33 [6, 30–32].

The Hungarian version [33] of the PHQ-15 [34] was used to evaluate the intrusiveness of somatic symptoms and a tendency toward somatization. The assessment tool contains 15 items, each addressing a frequently occurring mild, physical symptom such as back pain or trouble sleeping. Each item is scored on a 3-point scale ranging from 0 (not bothered at all) to 2 (bothered a lot). Internal consistency of the PHQ-15 was very good in the present sample (Cronbach’s alpha = 0.85).

Subjective psychological stress level was measured by the 4-item, Hungarian version [35] of the Perceived Stress Scale [36]. Each item is scored on a 5-point scale ranging from 0 (never) to 4 (very often). Internal consistency of this scale was excellent (Cronbach’s alpha = 0.82) in the present sample. Finally, the Hungarian, 5-item version [37] of the WHO Well-being Index [38] was used to measure participants’ overall subjective well-being. Items of this instrument are scored on a 4-point scale ranging from 0 (not at all characteristics of me) to 3 (very characteristic of me). Internal consistency of the scale was excellent in the present study (Cronbach’s alpha = 0.87).

### Statistical analyses

All statistical analyses were carried out using the SPSS 28® software (SPSS, Chicago, IL, USA). The sample was weighted according to the Deming-Stephan iterative proportionality fit model (raking method) [39] fitted to the data of 3,877,000 people (Hungarian working population). The relationship between the occurrence of offensive behaviours and the sociodemographic variables was assessed using the chi square test. Effect size was expressed using Cramer’s V and interpreted also considering the degrees of freedom [40].

The inspection of the continuous variables did not indicate severe deviation from the normal distribution (skewness ≤ 0.85, kurtosis ≤|0.42|) and therefore, the parametric univariate analyses of variance was used to investigate the relationship between offensive behaviour exposure and these variables (depressive symptomatology, somatic symptoms, perceived stress and general wellbeing). Effect size was expressed by eta squared, in which case 0.01 was considered as the threshold for small effect, 0.06 for moderate effect, and 0.14 for large effect [41].

## Results

**Fig. 1:**
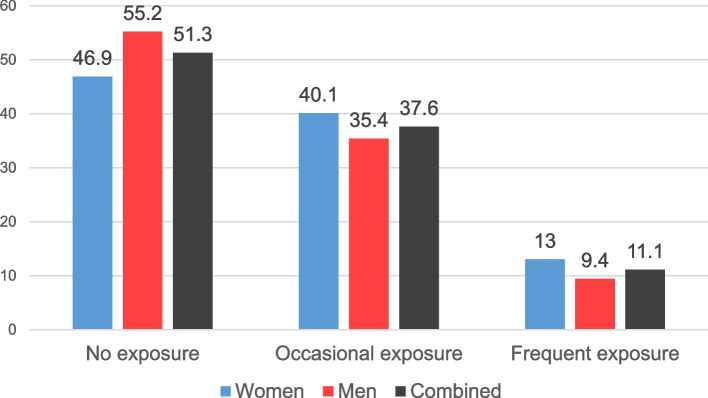
Gender differences in the 12-month prevalence (%) of exposure to workplace offensive behaviours

Almost half (48.7%) of the sample reported exposure to offensive behaviours in their workplace in the 12 months preceding the survey; 37.6% reported occasional exposure, while 11.1% of the respondents reported being the victim of offensive behaviours frequently (on a weekly or more frequent basis). The 12-month prevalence of exposure to each offensive behaviour is displayed in Table 2. The prevalence of offensive behaviour exposure was significantly but weakly associated with respondent gender (χ^2^ = 101.7, *p* < 0.001, Cramer’s V = 0.09): both occasional and frequent exposure was more prevalent in women (Fig. 1).

**Table 2:** 12-month prevalence of exposure to workplace offensive behaviours

	Bullying		Unwanted sexual attention		Threats of violence		Physical violence	
	*N*	%	*N*	%	*N*	%	*N*	%
No exposure	7,514	57.6	11,932	91.5	11,559	88.7	12,780	98.3
A few times	3,789	29.0	827	6.3	1,142	8.8	183	1.4
Monthly	548	4.2	84	0.6	124	0.9	11	0.1
Weekly	781	6.0	136	1.0	125	1.0	24	0.2
Daily exposure	423	3.2	68	0.5	80	0.6	7	0.1

**Fig. 2:**
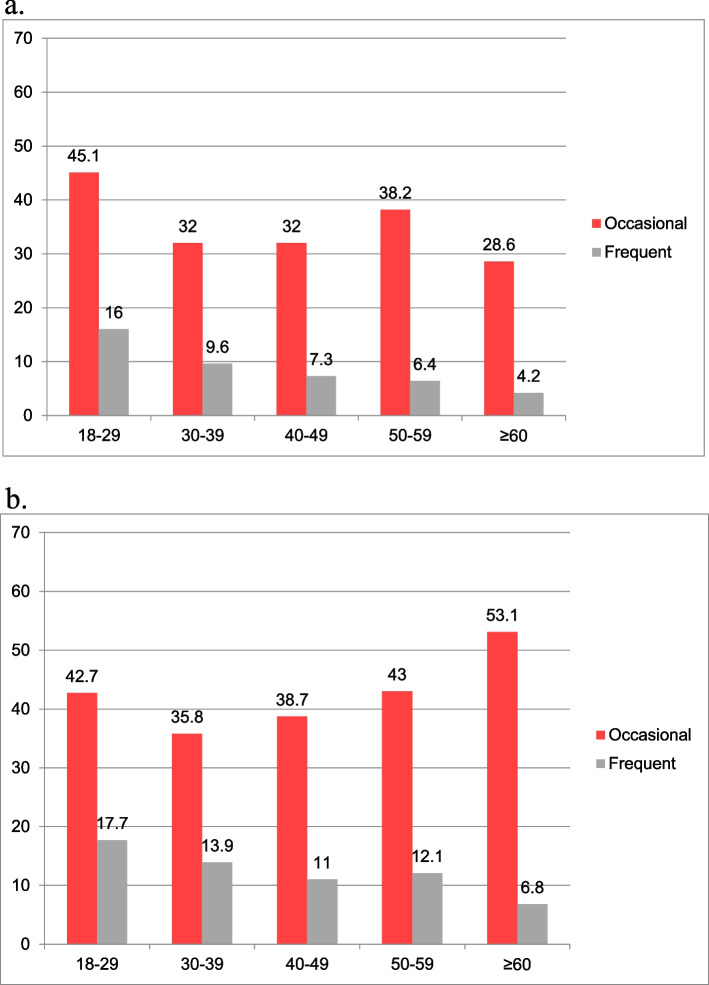
**a **Age differences in the 12-month prevalence (%) of occasional or frequent exposure to offensive behaviours in men. **b **Age differences in the 12-month prevalence (%) of occasional or frequent exposure to offensive behaviours in women

Similarly, the prevalence of exposure to offensive behaviours at the workplace was significantly and weakly associated with the age group of the respondents (χ^2^ = 242.5, *p* < 0.001, Cramer’s V = 0.10). Frequent exposure was most prevalent in the youngest (18–29 years) age group, independent of gender. However, occasional exposure was most prevalent in the oldest age group in women and the youngest age group in men (Fig. 2a, b).

**Fig. 3:**
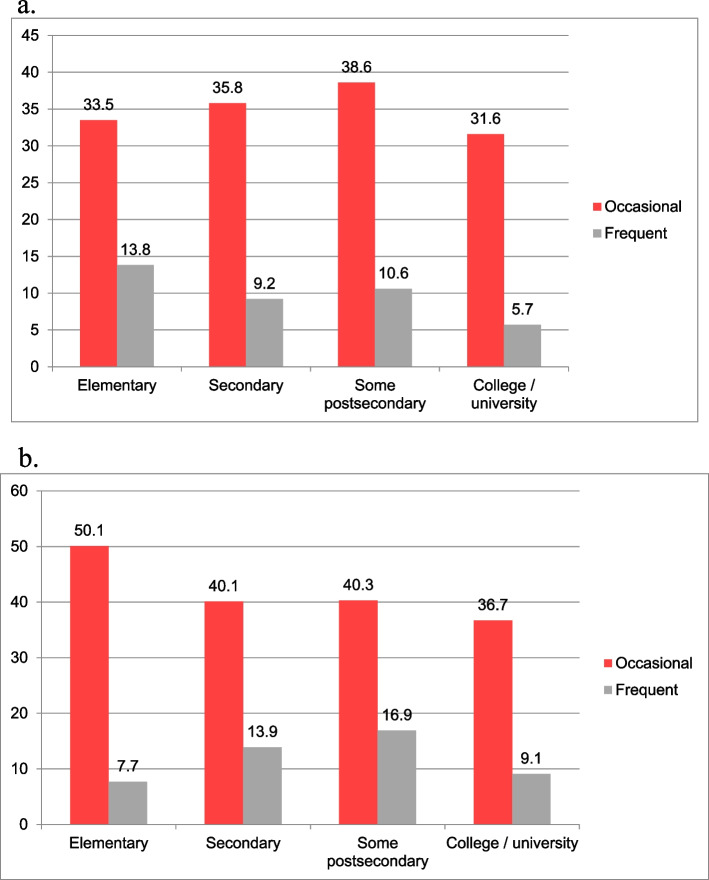
**a **Relationship between educational attainment and 12-month prevalence (%) of occasional or frequent exposure to offensive behaviours in men. **b** Relationship between educational attainment and 12-month prevalence (%) of occasional or frequent exposure to offensive behaviours in women

Exposure to workplace offensive behaviours was also significantly but weakly associated with the educational attainment of the respondents (χ^2^ = 141.5, *p* < 0.001, Cramer’s V = 0.07). In the case of both genders, those with the highest level of education were those who were least often exposed to any offensive behaviours. Nevertheless, occasional exposure was most prevalent in women with the lowest level of education, while interestingly in men, those with some postsecondary education were at highest risk (Fig. 3a, b). The trend was the exact opposite for frequent exposure to offensive behaviours, where those with some postsecondary education were at the highest risk in women and those with elementary education in men.

**Fig. 4:**
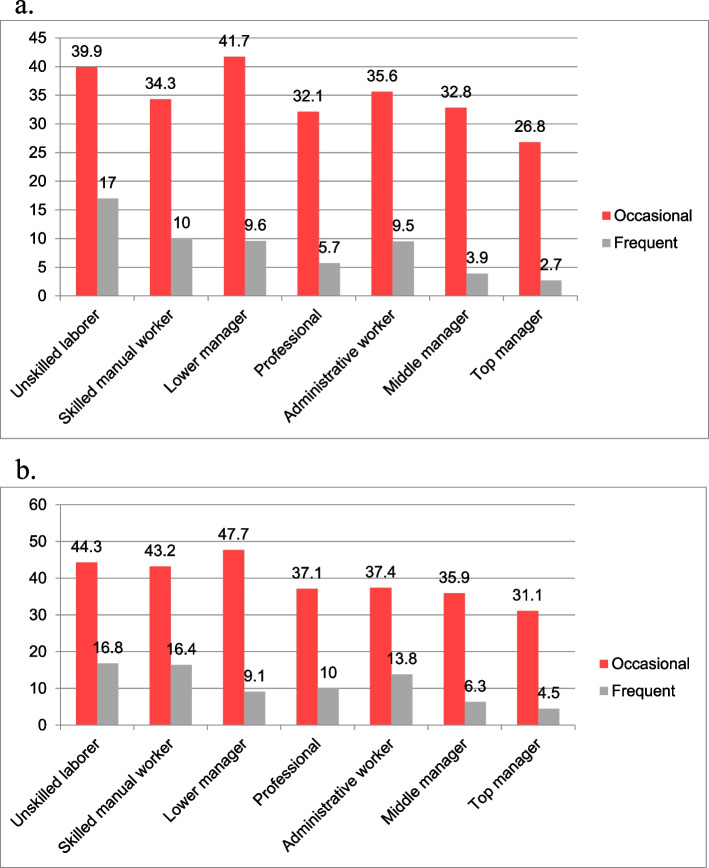
**a **Relationship between job position and 12-month prevalence (%) of occasional or frequent exposure to offensive behaviours in men. **b** Relationship between job position and 12-month prevalence (%) of occasional or frequent exposure to offensive behaviours in women

Offensive behaviour exposure was significantly associated with the job position of the victims as well (χ^2^ = 303.8, *p* < 0.001, Cramer’s V = 0.11) with the effect size hovering between the small and moderate range. In both genders, employees in lower level management positions experienced the highest prevalence of occasional exposure to offensive behaviours, while unskilled labourers were most often victims of offensive behaviours on a frequent basis (Fig. 4a, b).

**Fig. 5:**
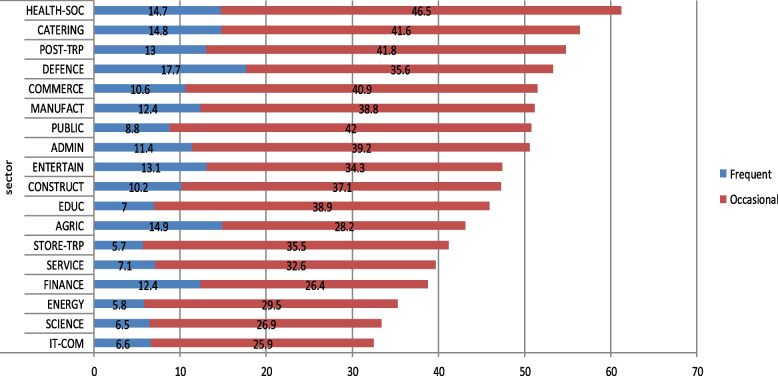
Relationship between industry sector and 12-month prevalence (%) of occasional or frequent exposure to offensive behaviours. ADMIN: Administrative and support service; AGRIC: Agriculture; forest; game and fisheries management; CATERING: Accommodation and food service; COMMERCE: Wholesale and retail trade; repair of motor vehicles; real estate; CONSTRUCT: Construction; DEFENCE: Defence (jurisdiction; military; fire service); EDUC: Education; ENERGY: Energy; mining; water and waste management; ENTERTAIN: Arts; entertainment; sport and recreation; FINANCE: Financial and insurance activities; HEAL-SOC: Human health; social work activities; IT-COM: IT and communication; MANUFACT: Manufacturing; POST-TRP: Passenger transport; postal services; PUBLIC: Public administration; social security; SCIENCE: Professional, scientific and technical activities; SERVICE: Other services (politics; NGOs, repair; beauty; undertaking etc.); STORE-TRP: Transportation and storage

We also examined if two organization-level variables – namely sector and company size – were associated with the prevalence of workplace offensive behaviours. The data revealed that the prevalence of offensive behaviours at the workplace was not independent of the sector of the company (χ^2^ = 328.3, *p* < 0.001, Cramer’s V = 0.11); frequent exposure was most common in the defence sector, while occasional exposure was most prevalent in the health and social care sector. Exact prevalence rates for both occasional and frequent exposure to offensive behaviours for each sector are displayed on Fig. 5.

**Fig. 6:**
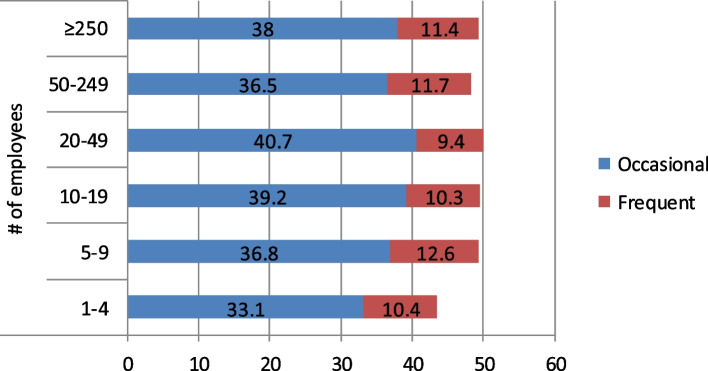
Relationship between company size and 12-month prevalence (%) of occasional or frequent exposure to offensive behaviours

Company size, defined by the number of employees, was also significantly but weakly associated with the occurrence of workplace offensive behaviours (χ^2^ = 30.8, *p* = 0.001, Cramer’s V = 0.03); both occasional exposure and the combined prevalence of occasional and frequent exposure was most prevalent in midsized companies employing 20–49 persons. Detailed data for these associations are displayed on Fig. 6.

In terms of the perpetrators of offensive workplace behaviours, 56.8% of the victims reported being offended by managers / superiors, 47.9% reported that co-workers committed these behaviours against them, 28.6% reported that perpetrators were clients / customers / patients, while 8.7% reported being victimized by subordinates (note that the same victim could indicate different types of perpetrators simultaneously).

The data also indicated that exposure to offensive behaviours at the workplace was moderately strongly associated with an increased level of depressive symptomatology, somatic symptoms, and perceived stress as well as decreased level of well-being (Table 3). The post hoc tests indicated significant differences between the three groups (no vs. occasional vs. frequent exposure to workplace offensive behaviours) across all mental health indicators (all *p*s < 0.001).

**Table 3:** Relationships between 12-month prevalence of exposure to workplace offensive behaviours and indicators of mental health

	No exposure†	Occasional exposure†	Frequent exposure†	Comparison of the groups
Depressive symptomatology	11.06 (10.92)	17.76 (12.0)	23.21 (12.83)	F = 886.9, *p* < 0.001, η^2^ = 0.12
Somatic symptoms	7.51 (5.19)	10.77 (5.49)	12.76 (5.57)	F = 846.2, *p* < 0.001, η^2^ = 0.12
Perceived stress	5.75 (3.08)	7.35 (2.88)	8.46 (2.83)	F = 706.3, *p* < 0.001, η^2^ = 0.10
General wellbeing	44.91 (21.6)	34.46 (19.95)	26.9 (18.6)	F = 636.5, *p* < 0.001, η^2^ = 0.09

^†^ Displayed values are means and standard deviations

## Discussion

Offensive workplace behaviours, defined by instances of bullying, sexual harassment, threats of violence, and physical violence, have been considered a serious risk to the health and safety of workers; however, the frequency and severity of these behaviours have varied extensively across studies and hardly any data are available from the Central-Eastern European region. Thus, the current study aimed to contribute to the body of knowledge surrounding offensive workplace behaviours by reporting the results of a large, representative study from Hungary catered to regional and international comparability. We also aimed to report data on the links between exposure to workplace offensive behaviours and mental health to contribute to the better understanding of the complex interrelationships between the workplace environment and health.

The lack of comprehensive data in Hungary prior to the current study has prevented evidence-based assessment of workplace bullying, yet it has not prevented opinions on its supposed prevalence and severity. In the first European Survey of Enterprises on New and Emerging risks, it was discovered that, in Hungary, more than 95% of health and safety representatives reported no concern about bullying in the workplace. Likewise, in Estonia, approximately 90% made similar claims, and in Slovenia, 0% perceived that these behaviours would pose a major concern, while only 5% reported some concern [42]. Despite these claims, evidence contrarily indicates that these countries are among the many other nations where workplace bullying is a concerning and prevalent risk [43, 44]. For instance, in the fourth European Working Conditions Survey, Slovenia was ranked 11th of the 31 countries surveyed into the prevalence of workplace bullying, with Estonia following in the 14th position [22]. The placement of these countries on the ranked list of the 2007 report is in stark contrast to the low risk perceived by managers and health and safety representatives interviewed in the subsequent 2010 report by the European Agency for Safety and Health at Work [42].

More recently, research has sought to ascertain a more accurate assessment of the problem in these countries, barring Hungary. To that end, Mumel and colleagues set out to investigate the relationship between offensive workplace behaviours and PTSD [43]. Their findings suggested, despite the reported low level of concern from Slovenian managers and health and safety representatives, that bullying was a serious problem, with as many as 63% of participants identified as occasional victims of bullying (experienced at least one negative act occasionally or monthly) and 24% as regular victims (experienced at least two negative acts weekly or more often). Interestingly, a supplementary assessment using the self-labelling method discovered that 36% of participants identified as occasional victims of bullying, while only 5% identified as regular victims.

Furthermore, in Estonia, Tambur and Vadi measured the prevalence of bullying and also identified the phenomenon as a serious problem, with occurrence rates as high as 8%, which is comparable to other countries where the prevalence of workplace bullying is considered to be high [44]. These findings seem to suggest a disconnect between health and safety personnel’s perception of the issue and evidence of its prevalence and severity. The findings of the current study demonstrate that workplace offensive behaviours pose a significant risk to the Hungarian workforce as well, and the evidence surrounding their health correlates suggests that they should be treated as such.

The current study revealed that almost half of the 13,104 participants had been exposed to some form of offensive behaviour in the workplace within a 12-month period, whereas 11.1% labelled themselves as frequent victims. The prevalence of these behaviours is comparable to a large meta-analytical study conducted by Nielsen and colleagues in 2010 [10]. In this study, a total of 102 estimates from 86 independent samples were included to establish a mean occurrence rate of 11.3%. In order to contribute to this pooled estimate, studies must have measured instances of workplace bullying using the self-labelling method with a definition provided and had to consist of the following operative criteria: "…bullying refers to situations where a person repeatedly and over a period of time is exposed to negative acts on the part of co-workers, supervisors or subordinates, and where the person confronted have difficulties defending himself/herself against the mistreatment" [10]. Due to the similarities between the current study's definition of workplace bullying and the recommendations by researchers and clinicians on how the term should be operationalized, our results can be said to be a valid comparison to the meta-analytical study and any study that employs the corresponding methodology.

Regarding the issue of gender, the prevalence of bullying in the present study was similar to other studies suggesting that women are somewhat more susceptible to being victimized than men [45]. On average, women experienced bullying more frequently than men on both on an occasional and a frequent basis (Occasional: 40.1% women, 35.4% men; Frequent: 13% women, 9.4% men), and men reported a higher frequency of no exposure compared to women (46.9% women, 55.2% men). As an estimate of the Hungarian working population, this means that approximately 277,000 women and 230,000 men experience frequent exposure to offensive workplace behaviours, based on the percentage of the total labour force (46.6% women, 53.4% men) [46] and February 2022 demographic of 4,569,400 Hungarian workers [47]. There was a 3.6% difference between women's (13%) and men's (9.4%) exposure, which is similar to other studies in Europe and abroad. For example, the government of Canada reported that 14% of women and 12% of men self-labelled as a victim of bullying in a national survey of public sector employees [48]. Likewise, in the United States, 28.5% of women and 25.2% of men self-labelled as a victim [49], and in Finland, 5.5% of women and 3% of men made similar claims [50]. That being said, several other studies have reported no gender-related differences in exposure and instances where men are somewhat more susceptible than women [45], and therefore, the issue remains somewhat inconclusive in the global context. Nonetheless, the current study supports the notion that, on average, women are somewhat more susceptible to exposure than men.

Increased levels of depressive symptomatology, somatic symptoms, perceived stress, and decreased levels of general well-being were revealed to be moderately strongly associated with exposure to workplace bullying in the current study. These findings are consistent with the consensus in the current literature that has revealed significant relationships between exposure to workplace bullying and psychological and psychosomatic complications. For instance, in a meta-analytical study by Verkuil and colleagues, exposure to workplace bullying was revealed to be moderately strongly associated with depression (*r* = 0.29, *p* < 0.001), anxiety and PTSD (*r* = 0.34, *p* < 0.001), and stress-related complaints (*r* = 0.37, *p* < 0.001) [51]. These findings were the result of a pooled estimate from 42 reports primarily covering data from North America and Europe and concluded, as our findings have, that exposure to bullying is a significant predictor of experiencing mental health-related complications. While the cross-sectional nature of the data does not allow firm conclusions to be made regarding causality, the overall pattern of the results may point into the direction that poorer mental health is rather the consequence than the cause of workplace offensive behaviours.

There has also been speculation and research surrounding the relationship between the victim's designation within their organization and susceptibility to bullying. However, findings have been inconclusive in this regard, too: some studies suggest a relationship between the prevalence of bullying and the victim's designation, stating that those in subordinate positions are at the highest risk of being subjected to offensive workplace behaviours, while other studies have reported results that contradict this claim [9]. The highest percentage of workers who reported no exposure to offensive workplace behaviours in the current study were those identified as top managers (67.6%). In contrast, the highest percentage of workers who reported frequent exposure were those identified as unskilled labourers (16.9%). Thus, the current study supports the notion that exposure to bullying is indeed related to one's workplace status, as reports of no exposure shared a positive-, while frequent exposure shared a negative directionality with organizational status.

More specifically, 56.8% of our respondents who reported being exposed to workplace bullying stated that these behaviours were perpetrated by their managers/superiors, while only 8.7% reported being bullied by their subordinates. Similar results were observed in another study that established support for the claim that managers are the most common perpetrators of bullying, with 77% of the reports having specified being bullied by their superiors [52]. In contrast, colleagues were identified as the most common perpetrators of workplace bullying in one Danish study, accounting for more than 70% of the reports [53], and another study in Denmark revealed that colleagues and managers were the most common perpetrators of bullying, with rates of 71.5% and 32.5%, respectively, while bullying from subordinates only accounted for 6% of the reports [54]. Indeed, the only value that most studies seem to have in common is a comparatively low percentage of those that experience bullying from their subordinates.

**Fig. 7:**
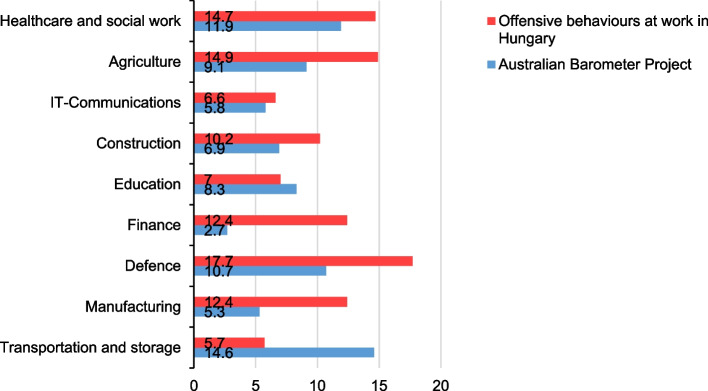
Industry-specific prevalence rates (%) of workplace offensive behaviours in Australia [21] versus Hungary (based on the results of the present study)

The industries whose workers reported the highest amount of frequent exposure to offensive workplace behaviours in the present study were defence (17.7%), agriculture (14.9%), and catering (14.8%), and those whose workers reported the highest amount of occasional exposure were health and social work (46.5%), passenger transport and postal service (41.8%), and catering (41.6%). Furthermore, those that reported the lowest amount of frequent exposure were employed in the transportation and storage (5.7%), energy (5.8%), and science (6.5%) sectors, while the lowest amount of occasional exposure was reported in IT and communications (25.9%), finance (26.4%), and science (26.9%). There are only few examples in the literature of representative studies that have examined the prevalence of workplace bullying across several occupational sectors. One of these is the Australian Barometer Project [21] providing sound comparative values due to the similarities in methodology and sampling strategies. In this study, the overall, national prevalence rate in 2015 was revealed to be 9.7%. Compared to the current study's prevalence of 11.1%, the presence of bullying in Australia and Hungary appears to be similar. That being said, some significant differences were observed in the prevalence of workplace bullying across occupational sectors. In particular, finance saw a difference of 9.7%, transportation and storage 8.9%, manufacturing 7.1%, and defence 7%, suggesting that sector-related estimates may vary considerably between countries (Fig. 7).

The relationship between culture and the prevalence of workplace bullying has received some significant consideration in the literature. It has been proposed that, in some countries, data reported through self-labelling methods may produce an inaccurate representation of the problem as this method relies on the participant's willingness to self-identify as a victim of a potentially taboo phenomena [55]. In order to investigate this issue, one comparative study examined the acceptability of bullying in the workplace across six continents and found that cultures that value results and performance typically perceive certain offensive behaviours as more acceptable than cultures that value diversity, loyalty, and sympathetic attitudes. Bullying seems to be most acceptable in Confucian Asia and least acceptable in Anglo, Latin American, and Sub-Saharan African countries [55].

While the present study has the major strength of being based on a large, nationally representative sample providing information on all major occupational sectors present in the Hungarian economy, the results also need to be seen in light of certain limitations. Most notably, the data collection period occurred well before the COVID-19 pandemic, which impacted workplace environments worldwide and saw a large number of workplace interactions shift into the virtual space. As a result, the pandemic may have impacted the prevalence of bullying as well (cf. the 43.2% prevalence rate of bullying victimization in the remote-, while 20.6% in the non-remote work setting in the US [56]), raising the possibility that the reported data are no longer entirely valid. That being said, the transition into the virtual setting was significantly less prevalent in Hungary than in many other industrialized nations due to shorter lockdown periods and less support for work-from-home accommodations. Nonetheless, the prevalence of workplace bullying could have been influenced by the pandemic, particularly in high-stress environments such as defence and health care, which would have seen significantly less work-from-home accommodations, increased absenteeism, staffing shortages, and higher volumes of stress. Consequently, future research should investigate how the COVID-19 pandemic might have influenced the prevalence and nature (e.g., virtual versus face-to-face interactions) of workplace bullying in Central/Eastern Europe both in the middle and the longer term. Researchers and clinicians should also consider the impact of methodological features on their outcomes (e.g., self-labelling vs. behavioural experience approach of assessment).

## Conclusions

The prevalence of frequent exposure to offensive workplace behaviours in the present, nationally representative Hungarian sample was 11.1%, using the self-labelling approach with definition. The groups most at risk for exposure to bullying were employees in health care/social work, catering, passenger transportation/postal services, defence, and commerce. When considering non-industry-specific variables, women, unskilled labourers, employees with some post-secondary education, workers aged 18–29, and individuals working for mid-sized companies are considered most at risk for workplace offensive behaviours. These results lend themselves to the growing body of evidence surrounding the prevalence of workplace bullying and serve as a valid regional and international reference for studies with a similar research design.

Increased levels of depressive symptomatology, somatic symptoms, perceived stress, and decreased levels of general well-being were moderately strongly associated with exposure to bullying. Thus, not only do the results indicate that bullying is a prevalent issue in Hungary, they further imply the potential for complications that can be detrimental to the health and safety of Hungarian workers. In light of this evidence, it is advisable that health and safety representatives acknowledge the presence of these offensive behaviours and employ strategies to reduce their occurrence and their impact on the work environment and worker health.

## Supplementary Information

**Additional file 1: Supplementary Table 1.** Occupational sector distribution of the sample

## Data Availability

The dataset generated and analyzed during the current study is not publicly available due to the lack of consent for public sharing from the sponsor of the study and the participants (consent for public data sharing was not sought in order to increase participation rate). However, the data may be shared by the corresponding author on an individual basis upon reasonable request.

## Footnotes

1 We chose the COPSOQ II for an assessment tool as it is a comprehensive instrument to assess the work environment in its complexity and its effect on health. This questionnaire includes 28 scales that cover – beyond exposure to offensive behaviours – both positive (e.g., collegial support, reward, fair treatment) and negative (e.g., fast work pace, emotional demands, role conflict) aspects of the work environment as well as health-related outcomes (e.g., burnout, sleep difficulties, self-rated health). These additional aspects we have analyzed and reported elsewhere [23].
