# Supplementary material for: The prevalence and mental health correlates of exposure to offensive behaviours at work in Hungary: results of a national representative survey
Source: BMC Public Health. 2023 Jan 11;23:78. doi: 10.1186/s12889-022-14920-0 (PMC9832257; doi:10.1186/s12889-022-14920-0)
Supplement: Supplementary file 1 — Additional file 1: Supplementary Table 1. Occupational sector distribution of the sample [file 12889_2022_14920_MOESM1_ESM.docx]

Supplementary Table 1. Occupational sector distribution of the sample

|  | **Started survey**  **N=19,280** | | **Included in final data set**  **N=13,104** | | **Weighted sample** |
| --- | --- | --- | --- | --- | --- |
|  | **N** | **%** | **N** | **%** | **%** |
| Agriculture, forest, game, and fisheries management | 257 | 1.3 | 164 | 1.3 | 5.2 |
| Energy, mining, water- and waste management | 421 | 2.2 | 274 | 2.1 | 2.8 |
| Manufacturing | 1237 | 6.4 | 1081 | 8.2 | 20.7 |
| Construction | 544 | 2.8 | 323 | 2.5 | 6.3 |
| Wholesale and retail trade, repair of motor vehicles, real estate | 1651 | 8.6 | 1134 | 8.7 | 14.6 |
| Transportation and storage | 576 | 3.0 | 388 | 3.0 | 3.9 |
| Accommodation and food service | 778 | 4.0 | 517 | 3.9 | 4.2 |
| IT and communication | 1748 | 9.1 | 1135 | 8.7 | 2.7 |
| Financial and insurance activities | 1281 | 6.6 | 902 | 6.9 | 2.4 |
| Professional, scientific, and technical activities | 1331 | 6.9 | 1000 | 7.6 | 3.4 |
| Administrative and support services | 530 | 2.7 | 427 | 3.3 | 3.4 |
| Public administration social security | 1097 | 5.7 | 814 | 6.2 | 4.5 |
| Education | 1968 | 10.2 | 1465 | 11.2 | 8.1 |
| Human health social work activities | 2445 | 12.7 | 1768 | 13.5 | 6.9 |
| Arts, entertainment, sport, and recreation | 533 | 2.8 | 377 | 2.9 | 1.6 |
| Other services (politics, NGOs, repair, beauty, etc.) | 913 | 4.7 | 635 | 4.8 | 2.4 |
| Defence (jurisdiction military fire service) | 654 | 3.4 | 422 | 3.2 | 3.9 |
| Passenger transport and postal services | 367 | 1.9 | 278 | 2.1 | 2.9 |
| Missing | 949 | 4.9 | 0 | 0.0 | 0.0 |
